# Replication stress by Py–Im polyamides induces a non-canonical ATR-dependent checkpoint response

**DOI:** 10.1093/nar/gku866

**Published:** 2014-09-23

**Authors:** Thomas F. Martínez, John W. Phillips, Kenneth K. Karanja, Piotr Polaczek, Chieh-Mei Wang, Benjamin C. Li, Judith L. Campbell, Peter B. Dervan

**Affiliations:** 1 Division of Chemistry and Chemical Engineering, California Institute of Technology, Pasadena, CA 91125, USA; 2 Braun Laboratories, California Institute of Technology, Pasadena, CA 91125, USA

## Abstract

Pyrrole–imidazole polyamides targeted to the androgen response element were cytotoxic in multiple cell lines, independent of intact androgen receptor signaling. Polyamide treatment induced accumulation of S-phase cells and of PCNA replication/repair foci. Activation of a cell cycle checkpoint response was evidenced by autophosphorylation of ATR, the S-phase checkpoint kinase, and by recruitment of ATR and the ATR activators RPA, 9-1-1, and Rad17 to chromatin. Surprisingly, ATR activation was accompanied by only a slight increase in single-stranded DNA, and the ATR targets RPA2 and Chk1, a cell cycle checkpoint kinase, were not phosphorylated. However, ATR activation resulted in phosphorylation of the replicative helicase subunit MCM2, an ATR effector. Polyamide treatment also induced accumulation of monoubiquitinated FANCD2, which is recruited to stalled replication forks and interacts transiently with phospho-MCM2. This suggests that polyamides induce replication stress that ATR can counteract independently of Chk1 and that the FA/BRCA pathway may also be involved in the response to polyamides. In biochemical assays, polyamides inhibit DNA helicases, providing a plausible mechanism for S-phase inhibition.

## INTRODUCTION

Many DNA-binding small molecules can challenge a cell's ability to accurately replicate its DNA. Tolerance to various forms of replication stress is possible with the aid of stress sensors and mediators that activate DNA repair and cell cycle pathways, collectively called the DNA damage response (DDR) (1). The master regulators of the DDR are ATR and ATM, two PI3 protein kinase family members, which respond to stalled replication forks and DNA breaks. ATR and ATM phosphorylate many substrates to stabilize the DNA replication fork and activate cell cycle checkpoints. The checkpoints slow cell cycle progression and allow time for the cell to respond to stress before entry into mitosis (2). During S-phase, ATR is recruited to sites of stalled replication by replication protein A (RPA)-bound single-stranded DNA (ssDNA) in the presence of DNA damage. ATR is activated by a complex of many proteins and phosphorylates a number of targets, among which Chk1, a cell cycle checkpoint kinase, is best understood (3,4). ATM is similarly recruited to sites of double-stranded breaks (DSBs) by the Mre11–Rad50–NBS1 complex, where it can phosphorylate Chk2, another cell cycle checkpoint kinase, and the histone variant H2AX (5). However, how the DDR reacts to specific types of stresses, what downstream signaling events are necessary and what physical structures are sensed is still under investigation (6). Furthermore, there are many levels of crosstalk between ATM and ATR and many targets beyond the checkpoint kinases, Chk1 and Chk2, which adds to the complexity (4). We have studied the checkpoint response activated by DNA minor groove binding pyrrole–imidazole (Py–Im) polyamides to discover what response polyamides elicit.

Py–Im polyamides are programmable small molecules that bind in the minor groove of double-stranded DNA (dsDNA) with affinities and specificities comparable to DNA-binding proteins (7,8). Binding of the polyamides alters the local helical structure of DNA (9). Eight-ring hairpin polyamides are cell permeable and localize to the nucleus in live cells (10). Py–Im polyamides are derived from the natural products distamycin A and netropsin (11). Distamycin A is cytotoxic at relatively high concentrations (12) and inhibits the activity of RNA polymerase, DNA polymerase, topoisomerases I and II and helicases (13–15). Previously, we showed that hairpin Py–Im polyamides designed to bind the androgen response element (ARE) decrease the expression of prostate cancer-related genes, inhibit RNA polymerase activity, upregulate p53 and induce apoptosis (16,17). Curiously, no evidence of DNA breaks was observed which usually occurs upon treatment with DNA damaging agents such as doxorubicin. However, effects on replication remain to be investigated.

Here we report that hairpin Py–Im polyamides targeted to the ARE cause replication stress, resulting in an accumulation of S-phase cells. Furthermore, the polyamide-induced checkpoint response activates ATR and downstream phosphorylation of the mini-chromosome maintenance complex (MCMs), but not the downstream ATR effector kinase Chk1. The checkpoint response also results in monoubiquitination of the Fanconi anemia/breast cancer (FA/BRCA) gateway protein FANCD2. The checkpoint is activated despite low levels of ssDNA formation and the absence of observable DNA breaks. We also show that polyamides are potent inhibitors of helicase unwinding *in vitro*, suggesting a model in which polyamides preclude fork progression through DNA binding. These results demonstrate that polyamides are capable of imposing replication stress and can activate both a non-canonical Chk1-independent ATR-checkpoint response and the FA/BRCA pathway, resulting in S-phase delay.

## MATERIALS AND METHODS

### Chemicals and reagents

Hairpin Py–Im polyamides **1** and **2** were synthesized on solid phase Kaiser oxime resin using previously published protocols (18). Gemcitabine, etoposide, hydroxyurea (HU) and doxorubicin were purchased from Sigma-Aldrich, as were all other reagents unless otherwise noted.

Antibodies purchased from Santa Cruz Biotech were: mouse anti-PCNA, anti-Chk1, anti-RPA2, anti-Rad17, anti-FANCD2, goat anti-ATR and rat anti-BrdU (CldU cross-reactivity). Antibodies purchased from Bethyl were: rabbit anti-H2AX, anti-MCM2, anti-MCM2pS108 and anti-RPA2pS4/S8. Antibodies purchased from Abcam were: rabbit anti-FANCD2, anti-MCM2pS108, anti-Rad9, anti-RPA2pS33, anti-Chk2 and anti-H2AXpS139. Antibodies purchased from Cell Signaling Technologies were: rabbit anti-ATMpS1981, anti-Rad17pS645, anti-Chk1pS345, anti-Chk1pS317 and anti-Chk1pS296. Rabbit anti-Chk2pT68 was purchased from Millipore. Rabbit anti-ATM was purchased from Calbiochem. Rabbit anti-ATRpT1989 was a gift of Prof. Lee Zou.

### Cell culture conditions

LNCaP, LNAR and DU145 cells were maintained in RPMI 1640 (Invitrogen) with 10% Fetal Bovine Serum (FBS) (Irvine Scientific) at 37°C under 5% CO_2_. LNCaP and DU145 cells were purchased from ATCC (Manassas, VA, USA). LNAR cells were a gift from C.L. Sawyers at Memorial Sloan-Kettering Cancer Center (New York, NY, USA).

### Cytotoxicity assay

IC_50_ values for cytotoxicity were determined using a sulforhodamine-based colorimetric assay for cellular protein content in 96-well microplates (19). LNCaP and LNAR cells were plated at 3000 or 4000 cells per well for the 72-h and 96-h time points, respectively. DU145 cells were plated at 2000 or 2500 cells per well. Polyamides were added in 100-μl RPMI 1640 supplemented with 10% FBS 24 h after plating. Quadruplicate wells were used for each concentration. Cells were fixed with 100-μl 10% trichloroacetic acid solution, washed, stained and dried as described. After solubilization of the bound dye in 10-mM Tris (pH 8), the absorbance was measured at 490 nm on a Victor microplate reader (PerkinElmer).

The cytotoxicity data are charted as a percentage of untreated controls, corrected for background absorbance. IC_50_ is defined as the concentration that inhibits 50% of control cell growth. These values were determined by nonlinear least-squares regression fit to *Y* = *A* + (*B−A*)/(1 + 10^∧^((Log EC50−*X*)*H, where *A* = max, *B* = min and *H* = Hill Slope. Three independent trials were averaged; stated IC_50_ values represent the mean and standard deviation. These calculations were performed using Prism 4 (GraphPad) software.

### Caspase 3/7 activation assay

DU145 cells were plated in 96-well microplates at 2000–8000 cells per well. As above, polyamides and controls were added 24 h after plating. Each time point was assayed in triplicate. At harvest, Caspase 3/7 activity was assessed using 100 μl of Caspase-Glo reagent (Promega), which contains the proluminescent caspase substrate DEVD-aminoluciferin. Luminescence was measured after 30-min incubation at room temperature. Luminescence data are expressed as a fold difference from untreated controls as measured using a Victor microplate reader (PerkinElmer).

The cell viability of each treatment condition was monitored in a sister plate using a tetrazolium-based assay for mitochondrial bioreductive capacity (20). Ten-microliter WST-1 reagent (Roche) was added to each well and incubated at 37°C for 30 min before measuring the absorbance at 450 nm. The WST-1 data are corrected for background absorbance and expressed as a percentage of untreated controls.

### PARP cleavage assay

400 000 DU145 cells were plated in 10-cm diameter dishes. Polyamides were added after 24 h and were allowed to incubate an additional 72 h. At harvest, cells were washed once with phosphate buffered saline (PBS) then treated with 400-μl ice-cold lysis buffer (20-mM Tris-HCl pH 7.5, 150-mM NaCl, 1-mM Na_2_ ethylenediaminetetraacetic acid (EDTA), 1-mM ethylene glycol tetraacetic acid (EGTA), 1% Triton, 2.5-mM sodium pyrophosphate, 2-mM β-glycerophosphate, 1-mM Na_3_VO_4_, 1-μg/ml leupeptin, 1-mM phenylmethylsulfonyl fluoride (PMSF)) for 5 min at 5°C. The lysate was sonicated for 15 s and then centrifuged for 10 min at 20 000 × g at 5°C. The supernatant was retained. Protein concentrations were determined by Bradford assay (Bio-Rad) using bovine serum albumin (BSA; Bio-Rad) to create a standard curve. PARP cleavage was assayed by sandwich ELISA (Cell Signaling Technology) and performed according to the manufacturer's recommendations. Ten-microgram total protein was loaded into each well of a microplate coated with anti-cleaved PARP (Asp214) mouse mAb and allowed to incubate overnight at 5°C. Rabbit anti-PARP mAb was then added, followed by anti-rabbit IgG conjugated to horseradish peroxidase. Triplicate wells were included for each condition, and the data are representative of both experimental replicates. The data are expressed as fold change from the untreated condition, showing the mean and standard deviation of each measurement.

### Cell cycle analysis

800 000 DU145 cells were plated in 10-cm diameter dishes for 24 h before treatment with polyamides for an additional 24 h. Cells were pulsed with 10-μM ethynyldeoxyuridine (EdU) 30 min before harvest to estimate the rate of DNA synthesis. Cells were trypsinized and pelleted at 300 × g with cell culture supernatant. Following overnight fixation in 70% ethanol, the cells were rehydrated in 1% BSA/PBS and processed with the Click-it EdU Alexa Fluor 488 Flow Cytometry assay kit (Invitrogen) using half the recommended A488 reagent. After overnight treatment with 0.2-mg/ml RNase A in 1% BSA/PBS, the cells were stained for DNA content with 7-aminoactinomycin D and analyzed on a FACSCalibur (Becton-Dickinson) instrument. The data were analyzed using FlowJo v9.5.3 (TreeStar) and are representative of two trials. Monoparametric, propidium iodide, flow cytometry was also used to evaluate the effect of polyamides **1** and **2** on cell cycle distribution. DU145 cells were treated with 1–100 μM of polyamide **1** or 0.1–10 μM of polyamide **2** for 48 h. The effect of PI3 kinases on cell cycle distribution was measured by treating DU145 cells with 10-μM polyamide **1** or 1-μM polyamide **2** as well as 2-mM caffeine, 4- or 10-μM NU6027 (Calbiochem) and 4- or 10-μM KU55933 (Calbiochem) for 36 h. Data were analyzed using FlowJo and fitted to the Watson (Pragmatic) model. The data are representative of two trials.

### Knockdown of ATR by siRNA

ATR was knocked down for cell cycle analysis and immunoblot experiments using 20-nM Silencer Select siRNA against ATR (Ambion, s536) and RNAiMAX lipofectamine (Life Technologies) according to the manufacturer's protocol. Twenty-nanomolar Silencer Select Negative Control #1 (Ambion) was used as a control. Briefly, the siRNA was incubated for 48 h, with a media swap after the first 24 h, prior to the addition of 10-μM polyamide **1** or 1-μM polyamide **2** for an additional 36 h. Efficiency of knockdown was determined by western blot.

### Proliferating cell nuclear antigen immunocytochemistry

Proliferating cell nuclear antigen (PCNA) immunocytochemistry experiments were performed as in (21). Briefly, DU145 cells were plated in 4-well glass chamber slides (Lab-Tek) at 70 000 cells per well. Polyamide **1** was added at a final concentration of 10 μM and polyamide **2** at a final concentration of 1 μM with 0.2% dimethyl sulfoxide (DMSO). After fixation, permeabilization and blocking, cells were incubated with mouse PCNA mAb at a 1:500 dilution at 4°C overnight. Cells were then washed, followed by incubation with Alexa Fluor 488-conjugated donkey anti-mouse IgG (Life Technologies) at a 1:400 dilution at room temperature for 2 h. Cells were washed and mounted with Prolong Gold Anti-Fade reagent with DAPI (Life Technologies). Images were obtained using a Zeiss LSM 510 Meta NLO with Coherent Chameleon and a Plan-Apochromat 63x 1.4-numerical aperture oil immersion objective lens and processed using the LSM Browser software package. Foci were counted using the open source Python software, FociCounter (http://focicounter.sourceforge.net/). Parameters were kept constant across all conditions for a particular replicate, but differed slightly over the three replicates to account for differences in staining. Cells that were likely positive but sufficiently out of focus so as to not produce distinct foci were not counted. The Kruskal–Wallis test was performed using Prism 4 (Graphpad) software.

### Assessment of phosphorylation of proteins by immunoblot

800 000 DU145 cells were plated in 10-cm diameter dishes and allowed to adhere for 24 h before treatment with 0.1% DMSO, polyamide **1** or polyamide **2** for the indicated time. Cells were lysed in TBS-Tx buffer (50-mM Tris-HCl pH 7.4, 150-mM NaCl, 1-mM EDTA, 1% Triton X-100) containing fresh protease inhibitors (Roche), 1-mM PMSF, phosphatase inhibitors and 10-mM N-ethylmaleimide. The samples were quantified by Bradford assay, denatured by boiling in Laemmli buffer, and total protein was separated by sodium dodecyl sulphate-polyacrylamide gel electrophoresis (SDS-PAGE) on 4–15% gradient polyacrylamide gels (Bio-Rad). After transfer to the nitrocellulose (Bio-Rad) or PVDF (Millipore) membrane and blocking with Odyssey Blocking Buffer (Li-Cor), primary antibodies were incubated overnight at 4°C. Donkey anti-rabbit, donkey anti-mouse or donkey anti-goat 800CW IR dye-conjugated secondary antibody (Li-Cor) was added and the bands were visualized on an Odyssey infrared imager (Li-Cor). For assessment of MCM2 and FANCD2 modification, plates treated with either DMSO, polyamide **1**, polyamide **2** or polyamide plus 2-mM caffeine, 10-μM KU55933 (KU, ATM inhibitor) or 10-μM NU6027 (NU, ATR inhibitor) were added together and harvested at the indicated times. For HU treatment, cells were incubated with the indicated inhibitor for 34 h prior to the addition of HU for the final 2 h before harvesting at 36 h. ATR immunoprecipitation was performed using pre-cleared Protein G agarose beads (Pierce) and either normal goat IgG or ATR (N19) antibodies (Santa Cruz) overnight at 4ºC. All immunoblots and accompanying quantifications are representative of at least two biological replicates.

### Chromatin fractionation assay

2 × 10^6^ DU145 cells were plated in 15-cm diameter dishes and allowed to adhere for 24 h, followed by treatment with 0.1% DMSO, 10-μM polyamide **1**, 1-μM polyamide **2** or 10-mM HU for indicated times. Chromatin fractions were prepared according to published protocols (22). Briefly, cells were harvested, washed with PBS and resuspended with 400-μl buffer A (10-mM HEPES pH 7.9, 10-mM KCl, 1.5-mM MgCl_2_, 0.34-M sucrose, 10% glycerol, 1-mM dithiothreitol (DTT) and fresh protease and phosphatase inhibitors). Triton X-100 was added to a final concentration of 0.1% and incubated on ice for 5 min followed by centrifugation at 1300 × g for 4 min to pellet the nuclei. Nuclei were washed with buffer A and then lysed with 400-μl buffer B (3-mM EDTA, 0.2-mM EGTA, 1-mM DTT) for 10 min on ice. Chromatin was pelleted by centrifugation at 1700 × g for 4 min. Isolated chromatin was washed once with buffer B and spun down at 10 000 × g for 1 min. The supernatant is completely removed and the chromatin pellet was resuspended in 300-μl SDS sample buffer and sheared for 20 s at 25% amplitude with a microtip adapter. Samples were then incubated at 80ºC and analyzed by SDS-PAGE and immunoblot.

### 5-chloro-2′-deoxyuridine immunocytochemistry

20 000 or 50 000 DU145 cells were plated in 4-well glass chamber slides and allowed to adhere for 24 h. Cells were incubated with 50-μM CldU for 48 h then the media was swapped and polyamide added. Following polyamide treatment, cells were washed, fixed with 2% formaldehyde (Ted Pella) and permeabilized with 0.2% Triton X-100. Following permeabilization, cells were blocked with 3% goat serum with 0.1% Triton for 45 min at room temperature. After blocking, cells were washed with 0.1% Triton and then incubated with rat anti-BrdU antibody (ICR1) at a concentration of 10 μg/ml in 3% goat serum for 30 min at 37ºC. After washes, cells were incubated with chicken anti-rat Alexa488 antibody at a concentration of 4 μg/ml. Finally, cells were washed and mounted then imaged as in PCNA staining above. Cells were scored as positive for ssDNA if >10 foci were counted. One hundred and fifty cells were counted per condition over three biological replicates.

### Single cell alkaline gel electrophoresis

The apparatus and reagent kit were purchased from Trevigen. The assay was performed according to the manufacturer's recommendations. Briefly, 800 000 DU145 cells were plated in 10-cm diameter dishes for 24 h before treatment with polyamides for an additional 36 h. Cells were harvested by trypsinization and washed once with cold PBS before being suspended in 37°C low-melting point agarose at 1 × 10^5^ cells/ml. An aliquot of the suspension was placed on a 37°C glass slide and allowed to cool for 30 min. The slides were bathed in lysis buffer for 30 min followed by a 30-min treatment with alkaline unwinding buffer (200-mM NaOH, 1-mM EDTA) at 5°C. The slides were subjected to electrophoresis at 21 V in a prechilled apparatus and fresh unwinding buffer for 30 min. The slides were washed twice in water and once in 70% ethanol, then dried for 30 min at 37°C. Dried slides were stained with 1X SYBR Gold in TE buffer for 30 min at room temperature, and excess dye was removed by blotting. Slides were dried and stored at room temperature with desiccant. Comets were visualized using a Zeiss LSM 510 Meta NLO confocal microscope with a 5x objective (Zeiss) and scored using Comet Assay IV image analysis software (Perceptive). A random sampling of 400 cells from two biological replicates was analyzed for each condition. The data are displayed as a box and whisker diagram showing median and middle quartiles with whiskers at the min and max.

### T7 gp4A helicase assays

Helicase assays using T7 gp4A (BioHelix) were performed as published in (23). First, 40 pmol of a 75-mer oligonucleotide was labeled with [γ-^32^P]ATP (MP Biomedicals) using T4 polynucleotide kinase (NEB) and annealed to 80 pmol of a 95-mer oligonucleotide with 56 complementary bases to form a forked substrate in STE buffer (100-mM NaCl, 10-mM Tris-HCl, 1-mM EDTA). The forked substrate was purified by extraction from a 10% non-denaturing acrylamide gel. To assess polyamide effects on gp4A helicase activity, the forked substrate (1:1000 dilution final) was incubated in a 10-μl volume with increasing concentrations of polyamide **1** (DMSO solution, 5% final concentration) in 1x reaction buffer (BioHelix) for 1 h at room temperature prior to addition of gp4A at a final concentration of 143 ng/ml (∼2.27 nM) and incubated at 30°C for 10 min. The mock-treated helicase reaction contained 5% DMSO with no polyamide. Reactions were stopped with the addition of 5-μl stop buffer (60-mm EDTA, 40% sucrose, 0.6% SDS, 0.25% bromphenol blue and 0.25% xylene cyanole FF). Unwound-labeled single-stranded 75 mer was separated from the intact fork substrate on a pre-run 10% non-denaturing acrylamide gel at 200 V for 1 h. The gel was then placed on a PhosphorImager screen (Molecular Dynamics) overnight and imaged on a Storm Molecular Imager. Match 75mer: 5′-CGC CGG GTA CCG AGC TCG AAT TCA CTG GCC GTC GTT TTA CAA CGT CG**T GAA CT**G CCT_19_-3′. Match 95mer: 5′-T_39_GGC **AGT TCA** CGA CGT TGT AAA ACG ACG GCC AGT GAA TTC GAG CTC GGT ACC CGG CG-3′. Mismatch 75mer: 5′-CGC CGG GTA CCG AGC TCG AAT TCA CTG GCC GTC GTT TTA CAA CGT CG**T GAC AT**G CCT_19_-3′. Mismatch 95mer: 5′-T_39_GGC **ATG TCA** CGA CGT TGT AAA ACG ACG GCC AGT GAA TTC GAG CTC GGT ACC CGG CG-3′.

### *S. cerevisiae* Dna2-K677R helicase assay

*Saccharomyces cerevisiae* K677R Dna2 (yDna2-K677R), lacking nuclease activity, was prepared and helicase assays were performed similar to (24). First, 40 pmol of a 42-mer oligonucleotide was labeled with [γ-^32^P]ATP (MP Biomedicals) and annealed to 80 pmol of a 29-mer oligonucleotide with 24 complementary bases to form a forked substrate in STE buffer. To assess polyamide effects on yDna2-K677R helicase activity, the forked substrate (1:2000 dilution final) was incubated in a 20-μl volume with increasing concentrations of polyamide **1** (DMSO solution, 5% final concentration) in 1x reaction buffer (25-mm Tris-HCl (pH 7.5), 2-mM MgCl_2_, 2-mm DTT, 0.25-mg/ml BSA, 2-mM adenosine triphosphate (ATP)) for 1 h at room temperature prior to addition of 150-fmol yDna2-K667R and incubated at 37°C for 30 min. The mock-treated helicase reaction contained 5% DMSO with no polyamide. Reactions were stopped with the addition of 5-μl stop buffer. Unwound-labeled single-stranded 42 mer was separated from the intact fork substrate on a pre-run 20% non-denaturing acrylamide gel run at 150 V for 3 h. The gel was then placed on a PhosphorImager screen overnight and imaged on a Storm Molecular Imager. Match 42mer: 5′-AGC TAG CTC TTG ATC GTG ACG **AGA ACA** CCA GAA CGA GTA GTA-3′. Match 29mer 5′-TAC TAC TCG TTC TGG **TGT TCT** CGT TGA TC-3′. Mismatch 42mer: 5′-AGC TAG CTC TTG ATC GTG ACG **AGA AAA** CCA GAA CGA GTA GTA-3′. Mismatch 29mer 5′-TAC TAC TCG TTC TGG **TTT TCT** CGT TGA TC-3′.

### *S. cerevisiae* Dna2-K677R ATPase assay

The ATPase assay was run as in (24). Briefly, reactions containing 300 fmol of yDna2-K677R protein in 20 μl of reaction buffer (40-mM Tris-HCl (pH 7.5), 5-mM MgCl_2_, 25-mM NaCl, 1-mM DTT, 0.5-mg/ml BSA, 0.2-mM ATP, 10% glycerol and 3 μCi of [γ-^32^P]ATP) were supplemented with the mismatch 42-mer plus DMSO or 3-μM polyamide **1** and incubated at 30°C for 1 h. The reactions were stopped by addition of EDTA. An amount of 0.8 μl of each reaction was spotted onto a polyethyleneimine-cellulose TLC plate (Selecto Scientific) and developed in 0.5-M LiCl, 1-M formic acid solution. The radiolabeled products were detected by PhosphorImager.

### Clonogenic assays

Clonogenic assays were performed with FANCD2-deficient PD20 cells complemented with empty vector (PD20-EV) or FANCD2 (PD20-FANCD2). FANCD2 protein expression and phenotype rescue was previously confirmed (25). Briefly, 1000 cells per well were seeded in a 12-well plate and left to attach overnight. Polyamide **1** (0, 10, 20, 30 μM) or polyamide **2** (0, 1, 2, 3 μM) was added for 36 h. Polyamide-containing media was exchanged for fresh media and cells were cultured for 14 days with media changed every 4 days. Visible colonies were fixed and stained with 1% crystal violet in methanol and enumerated. Three independent experiments were performed.

## RESULTS

### Py–Im polyamides cause accumulation of S-phase cells and PCNA foci

**Figure 1.**
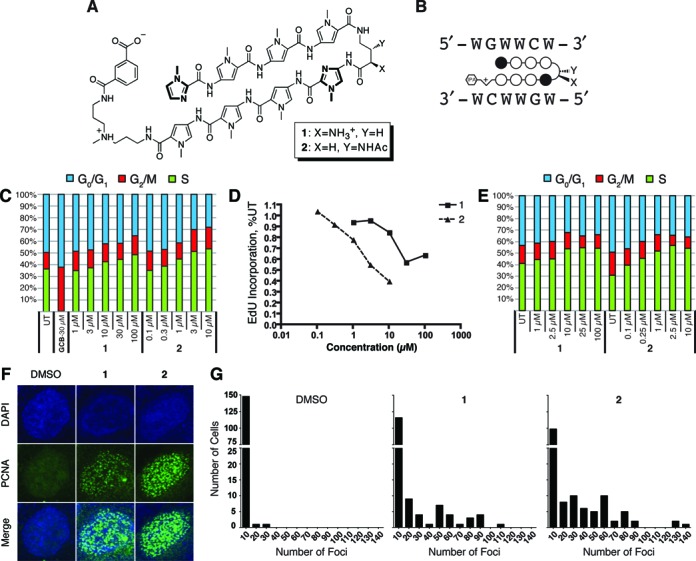
Polyamides cause accumulation of S-phase cells and PCNA foci. (**A**) Chemical structure Py–Im polyamides used in this study. (**B**) Ball-and-stick representation of the polyamides. Open circles represent N-methylpyrrole residues and filled circles represent N-methylimidazoles. The hexagon represents the isophthalic acid moiety. Polyamides **1** and **2** are specific for the same 5′-WGWWCW-3′ DNA sequence, where W = A or T. (**C**) Cell cycle distribution of DU145 cells untreated (UT) or treated with gemcitabine (GCB), polyamide **1** or polyamide **2** for 24 h as measured by two-color flow cytometric evaluation of EdU pulse-labeled cells stained for DNA content with 7AAD. (**D**) Dose-dependent decrease in average EdU incorporation indicative of slowed DNA synthesis in response to polyamide treatment. (**E**) Cell cycle distribution of DU145 cells untreated or treated with polyamide **1** or **2** for 48 h as measured by single-color flow cytometric evaluation of propidium iodide stained cells. (**F**) Representative images of immunofluorescent detection of PCNA in DU145 cells. Treatment with either 10-μM polyamide **1** or 1-μM **2** for 36 h causes more cells to contain significant punctate staining of PCNA. (**G**) PCNA foci counts for each cell are plotted in a histogram with bin sizes of 10 foci for each condition. One hundred and fifty cells over three replicates were counted for each condition. The Kruskal–Wallis test reports *P* < 0.0001 for **1** versus DMSO and **2** versus DMSO.

Hairpin Py–Im polyamides **1** and **2** were designed to target the ARE (5′-GGTACANNNTGTTCT-3′ (26)) and antagonize gene expression changes driven by the androgen receptor (AR) in the prostate cancer cell line, LNCaP (Figure 1A and B) (16,27). In LNCaP cells, AR signaling plays a critical role in cell proliferation (28), therefore disruption of AR-dependent signaling may contribute to cell death. However, disruption of other DNA-dependent processes such as RNA pol II transcription elongation may also cause cell death. To investigate the effects of polyamides outside of AR-dependent transcription, we first compared the cytotoxicity of polyamides **1** and **2** in three different prostate cancer cell lines, LNCaP, LNAR and DU145, which express high, normal and low levels of AR, respectively. Polyamides **1** and **2** displayed dose-dependent cytotoxicity at 72 and 96 h as measured by sulforhodamine B staining (Supplementary Table S1). Polyamide **2** had ∼10-fold higher potency than polyamide **1**, which is consistent with its greater potency against AR-driven gene expression (27). Importantly, the IC_50_ values were similar in all cell lines regardless of AR status, suggesting that the observed cytotoxicity occurred via an AR-independent mechanism. In DU145 cells, expression of an AR-driven reporter is insensitive to androgen treatment and AR is minimally expressed (29). Therefore, DU145 cells provide an environment to investigate the effects of polyamides **1** and **2** independent of AR signaling.

Next, we examined the effects of polyamides **1** and **2** on the cell cycle in DU145 cells. We pulse-labeled exponentially growing and asynchronous DU145 cells with EdU after 24 h of polyamide treatment. Both polyamides produced a dose-dependent increase in the percentage of cells in S-phase, with a corresponding drop in the percentage of G0/G1 cells (Figure 1C). Although more cells were in S-phase, the average intensity of EdU staining decreased, suggesting that the treated cells were replicating their DNA more slowly and thus cells spent longer in S-phase (Figure 1D). Similar results were also obtained using traditional one-color flow cytometry to determine the cell cycle distribution after 48 h of polyamide treatment (Figure 1E).

We then determined whether replication/repair foci accumulated in the treated cells using PCNA immunofluorescence (21,30). We chose treatment conditions to allow for maximal effect on the cells before any significant decrease in viability or activation of apoptosis, as measured by mitochondrial reduction activity and caspase 3/7 activation (Supplementary Figure S1). Nearly all DMSO-treated cells showed 0–2 foci per cell, while polyamide treatment resulted in a significant increase in cells with greater than 20 foci (Figure 1F and G). Interestingly, some of the polyamide-treated cells but none of the DMSO-treated cells showed more than 50 foci. Observation of cells with such high incidence of foci suggests that polyamides cause prolonged stalling of replication forks and the recruitment of repair machinery (30).

### Py–Im polyamide treatment induces ATR activation

**Figure 2.**
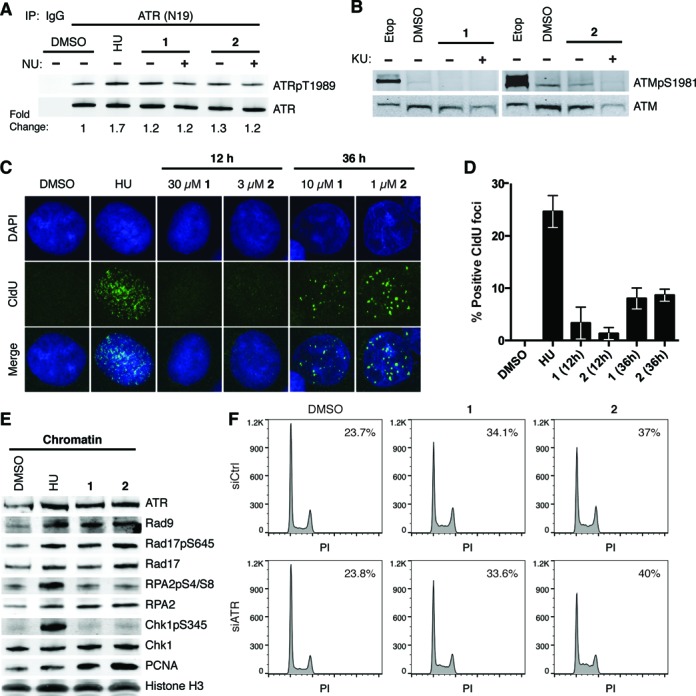
Polyamides induce ATR activation without extensive ssDNA formation. (**A**) Immunoblot of ATRpT1989 and ATR following immunoprecipitation (IP) of ATR in DU145 whole cell lysates treated with 4-mM hydroxyurea (HU) for 2 h, and DMSO, 10-μM polyamide **1** or 1-μM polyamide **2** in the presence or absence of 10-μM NU6027 (NU, ATR inhibitor) for 36 h. (**B**) Immunoblots of ATMpS1981 and ATM after treatment with 30-μM etoposide (Etop) for 30 min, and DMSO, 10-μM polyamide **1** or 1-μM polyamide **2** in the presence or absence of 10-μM KU55933 (KU, ATM inhibitor) for 36 h. (**C**) Representative images of ssDNA formation via CldU immunofluorescence under non-denaturing conditions are shown for cells after treatment with 4-mM HU for 2 h, DMSO, 30-μM polyamide **1** or 3-μM polyamide **2** for 12 h, and 10-μM polyamide **1** or 1-μM polyamide **2** for 36 h. (**D**) Bar graphs of the mean and standard deviation of percent CldU positive cells (>10 foci/cell). One hundred and fifty cells over three replicates were counted for each condition. (**E**) Immunoblots of ATR and checkpoint-related factors loaded onto chromatin upon treatment with 10-mM HU for 2 h, and DMSO, 10-μM polyamide **1** or 1-μM polyamide **2** for 36 h. (**F**) DNA histograms of propidium iodide (PI) stained DU145 cells after treatment with negative control or ATR-targeting siRNA for 48 h followed by treatment with DMSO, 10-μM polyamide **1** or 1-μM polyamide **2** for 36 h. The percentage of cells in S-phase is included at the top right of each graph.

S-phase accumulation subsequent to treatment with a DNA-binding compound was suggestive of checkpoint activation in response to replication stress. We therefore probed for activation of the master regulator kinases, ATR and ATM. We assayed ATR activation by immunoblotting for T1989 phosphorylation, an autophosphorylation site that has been implicated in ATR activation and a robust checkpoint response (31,32). Cells treated with polyamide **1** or **2** showed a slight increase in ATR T1989 phosphorylation relative to DMSO-treated cells (Figure 2A). However, cells treated with HU, which causes nucleotide depletion, showed greater ATR T1989 phosphorylation compared to polyamide-treated cells suggesting a weaker activation of ATR by polyamides. NU6027, which inhibits cellular ATR but not ATM, did not abrogate T1989 phosphorylation under polyamide treatment (33). While polyamide treatment appeared to activate ATR, polyamides did not induce ATM S1981 phosphorylation, an autophosphorylation site that has been associated with ATM activation and stabilization at DSBs (Figure 2B) (34).

The weak phosphorylation of ATR suggested that polyamide treatment might result in limited ssDNA formation (4). To directly probe for ssDNA accumulation, we preincubated cells with the thymidine analog, 5-chloro-2′-deoxyuridine (CldU), and then treated with polyamide or HU. After treatment, we fixed the cells and immunostained using an anti-CldU antibody, which reacts with CldU exposed in ssDNA but not dsDNA. About 25% of cells on average showed >10 CldU foci after treatment with HU, while only about 3% and 1% of cells showed >10 CldU foci after 12-h treatment with high concentrations of polyamide **1** or **2** (Figure 2C and D). When treated with lower concentrations of polyamide **1** or **2** for 36 h post-CldU incubation, about 8% and 9% cells were positive for CldU foci. However, among the positive cells present under polyamide treatment the number of CldU foci was substantially lower than in HU-treated cells. Thus, the degree of ssDNA formation in polyamide-treated cells was also lower than in HU-treated cells, consistent with the lower levels of T1989 phosphorylation observed.

To confirm ATR activation, we determined if ATR and mediators of the ATR response accumulate on chromatin after polyamide treatment. Polyamide treatment resulted in ATR loading onto chromatin (Figure 2E). Interestingly, although we had observed a lower level of ATR phosphorylation in polyamide-treated cells than in HU-treated cells (Figure 2A), similar amounts of ATR were loaded onto chromatin following each treatment. Polyamide treatment also induced loading of RPA, the Rad9–Rad1–Hus1 (9-1-1) complex, which is integral to ATR checkpoint signaling, and Rad17, which is part of the clamp loader that facilitates 9-1-1 loading, to similar levels as did HU. Rad17 S645, a target for ATR phosphorylation that is necessary for G2 checkpoint activation (35), was phosphorylated in the presence of polyamide, indicating that ATR was activated. Polyamide treatment also induced higher PCNA loading on chromatin, which is consistent with the high incidence of PCNA foci formation (Figure 1D). Despite the lack of extensive ssDNA formation, ATR as well as its mediators is recruited to chromatin and ATR is active after polyamide treatment.

### Py–Im polyamide-induced S-phase delay is not abrogated by ATR knockdown

To determine whether the activation of ATR had physiological consequences, we monitored the effect of siRNA knockdown of ATR on accumulation of polyamide-treated cells in S-phase. The percentage of S-phase cells was the same in cells treated with either siRNA against ATR or negative control siRNA prior to treatment with polyamide **1** or **2** (Figure 2F). This suggests that ATR activity is not contributing to S-phase accumulation. When caffeine, a PI3 kinase inhibitor with preference for ATR over ATM, was added to cells in addition to polyamide **1** or **2**, the S-phase population was reduced compared to cells treated only with polyamide; however, caffeine treatment also reduces the basal level of S-phase cells and may account for this decrease (Supplementary Figure S2). Similarly, when the ATR inhibitor NU6027 was added to cells the S-phase population was reduced under both the basal and polyamide-treated conditions.

Although ATM S1981 was not phosphorylated in response to polyamide treatment, ATM autophosphorylation sites other than S1981 have been implicated in its activation and function in the cell cycle checkpoint (36). Therefore the effects of ATM inhibition were also monitored. KU55933, a selective inhibitor of ATM, did not diminish the polyamide-induced S-phase accumulation (Supplementary Figure S2) (37).

### Py–Im polyamide treatment does not induce Chk1, RPA2 or Chk2 phosphorylation

**Figure 3.**
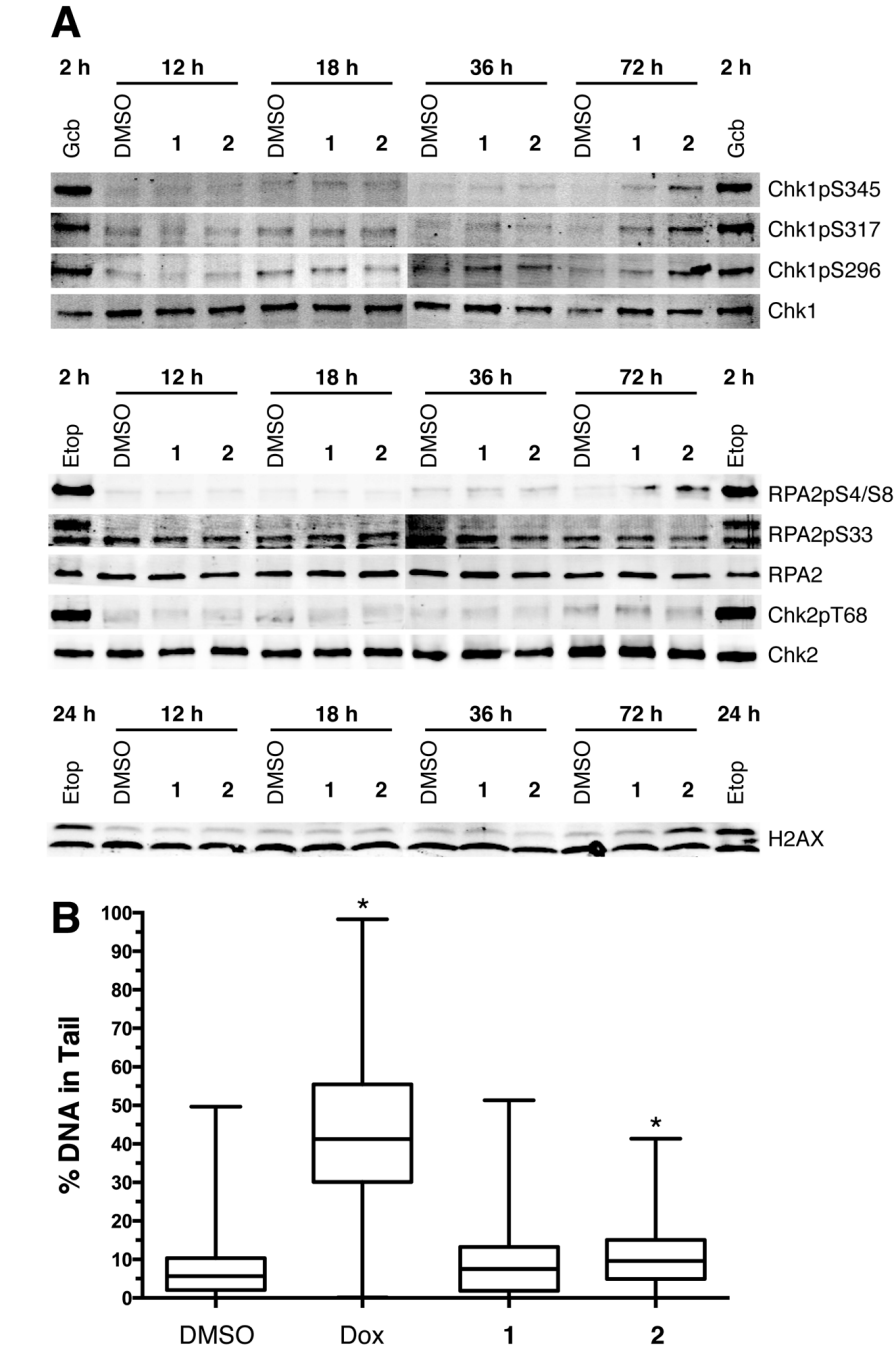
Polyamides do not induce phosphorylation of Chk1, RPA2 or Chk2 or observable DNA breaks. (**A**) Immunoblots of phosphorylated Chk1 at S345, S317 and S296; RPA2 S4/S8 and S33; Chk2 T68; and H2AX S139 after 12, 18, 36 and 72 h treatment with DMSO, 10-μM polyamide **1**, 1-μM polyamide **2**, or treatment with 30-μM gemcitabine (Gcb) for 2 h or 30-μM etoposide (Etop) for 2 h and 24 h in whole cell lysates. (**B**) Single cell alkaline gel electrophoretic analysis DU145 cells treated with 1-μM doxorubicin (Dox) for 24 h, and DMSO, 10-μM polyamide **1** or 1-μM polyamide **2** for 36 h. Boxes show the median percentage of DNA in the comet tail and are bounded by 25th and 75th percentile while whiskers represent the min and max percentile. Four hundred cells from two biological replicates were counted for each condition. The Mann Whitney test reports *P* < 0.0001 for Dox and **2**, and is indicated by *.

The ATR-mediated checkpoint response can be propagated by a variety of downstream effectors. Chk1, the best studied of the ATR effectors, signals cell cycle delay after activation by ATR via phosphorylation at S345. Surprisingly, Chk1 S345 was not phosphorylated after treatment with polyamide (Figure 2E). Chk1 S345 phosphorylation is dependent upon RPA2 hyperphosphorylation at sites S4 and S8, which occurs following DSBs from collapsed replication forks (38). Polyamide treatment also did not induce phosphorylation of RPA2 S4/S8. To ensure that we were not missing a transient activation of Chk1 or RPA2, we assayed for their phosphorylation across multiple time points. In addition, we monitored other known Chk1 and RPA2 phosphorylation sites including Chk1 S317 and S296 and RPA2 S33. Chk1 S317 is another target for phosphorylation by ATR in response to replication stress, and Chk1 S296 is an autophosphorylation site that is important for its function (39). RPA2 S33 phosphorylation by ATR under replication stress protects cells by stimulating DNA synthesis and facilitates S4/S8 phosphorylation by DNA-PKcs (38,40). After 12, 18, 36 and 72 h of treatment with polyamide **1** or **2**, neither Chk1 nor RPA2 was phosphorylated at any of the sites monitored (Figure 3A). To test the possibility that polyamides **1** and **2** may somehow inhibit ATR from phosphorylating Chk1, DU145 cells were treated with both polyamide **1** or **2** and aphidicolin, a DNA polymerase inhibitor that induces Chk1 S345 phosphorylation. The polyamides did not inhibit aphidicolin-induced Chk1 S345 phosphorylation (Supplementary Figure S3A and B).

The absence of Chk1 or RPA2 phosphorylation led us to investigate phosphorylation of Chk2, another cell cycle checkpoint kinase, and H2AX, a histone variant that is phosphorylated rapidly upon DNA damage, as possible downstream checkpoint mediators. ATM predominantly phosphorylates Chk2 T68, though there is evidence for phosphorylation of Chk2 by ATR following cisplatin treatment (41,42). Similarly, ATM or ATR can phosphorylate H2AX S139 in response to different types of replication stress (43). Consistent with the absence of ATM S1981 phosphorylation after polyamide treatment, polyamides failed to induce Chk2 T68 phosphorylation (Figure 3A). H2AX and RPA2 S4/S8 phosphorylation were slightly elevated after 72-h treatment of 1-μM polyamide **2**, and may be suggestive of DNA damage. However, H2AX can be phosphorylated under non-damaging stress (44). It is also worth noting that these phosphorylation events may be triggered by apoptosis, which occurs after 72-h treatment with polyamide **2** (Supplementary Figure S1). Finally, we also studied the effect of high concentration polyamide treatment for 18 h and similar results were observed (Supplementary Figure S3C).

### Py–Im polyamide treatment does not induce DNA breakage

The absence of ATM, Chk2 and RPA2 phosphorylation suggested that polyamide-induced replication stress does not lead to gross breakage of DNA. To study DNA breakage directly, we treated cells with polyamides and then analyzed them by single cell alkaline gel electrophoresis (Figure 3B). Migration of the DNA from the centroid into the ‘comet tail’ is proportional to the amount of single- and double-strand breakage that has occurred. Cells treated with doxorubicin, a known DNA-damaging agent, were used as a positive control and showed a median value of 41% of total DNA in the tail. Polyamide-treated cells, however, were similar to the DMSO control with median%DNA in tail values of 8 and 10 for polyamides **1** and **2**, respectively, compared to 6% for DMSO. The lack of extensive DNA breakage correlates with the absence of ATM-Chk2 activation.

### Py–Im polyamide treatment induces MCM2 phosphorylation and monoubiquitination of FANCD2, a major gatekeeper of the FA/BRCA repair pathway

**Figure 4.**
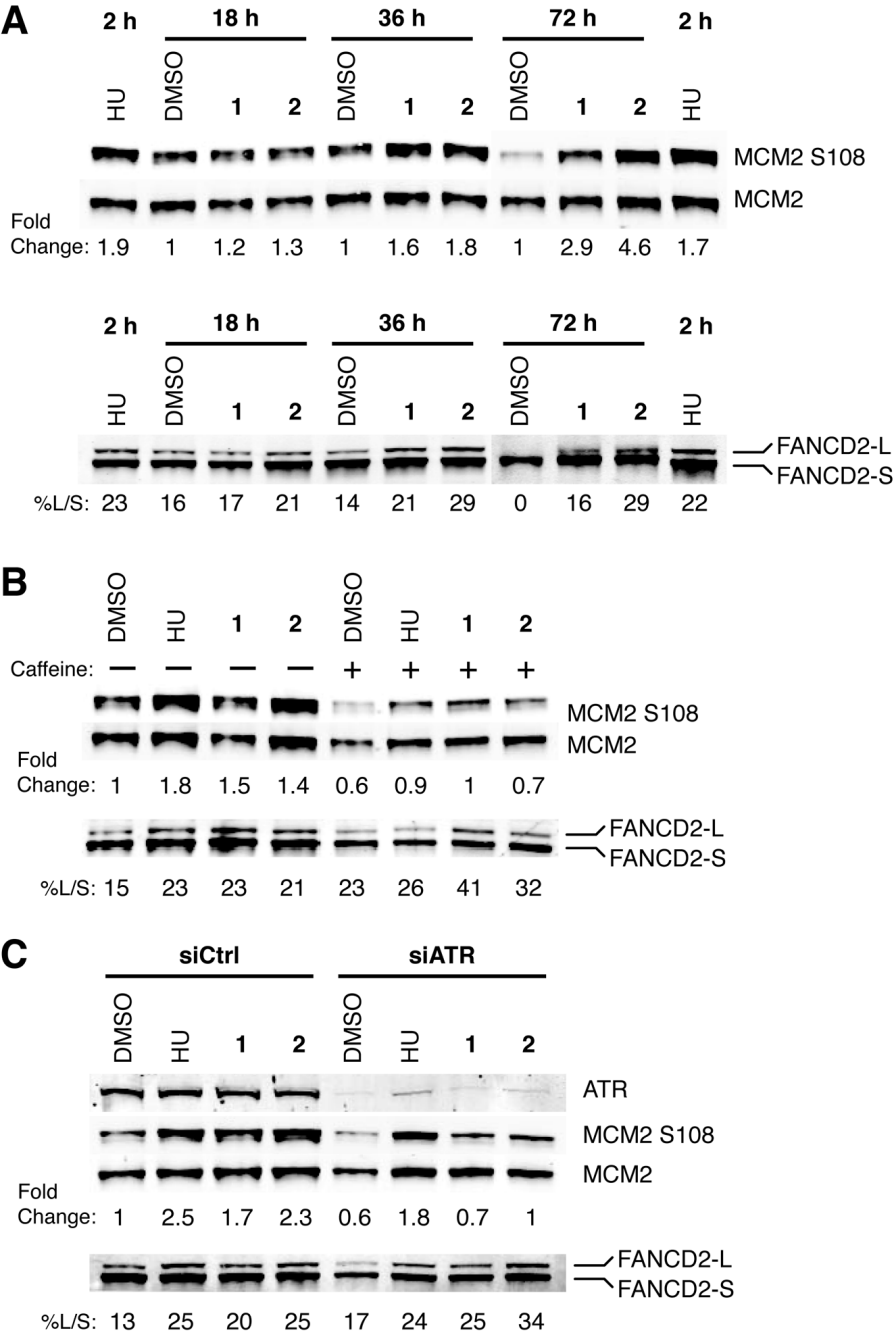
Polyamides induce phosphorylation of MCM2 and FANCD2 monoubiquitination. (**A**) MCM2 S108 phosphorylation and FANCD2 monoubiquitination levels were measured in DU145 cells treated with 4-mM HU for 2 h, and DMSO, 10-μM polyamide **1** or 1-μM polyamide **2** over a time course of 18, 36 and 72 h. Monoubiquitination was estimated by normalizing the band intensity of the large molecular weight monoubiquitinated FANCD2 band (FANCD2-L) to the low molecular weight non-ubiquitinated FANCD2 band (FANCD2-S). (**B**) MCM2 S108 phosphorylation and FANCD2-Ub levels were measured in cells treated with 4-mM HU for 2 h, and DMSO, 10-μM polyamide **1** or 1-μM polyamide **2** for 36 h with or without the addition of 2-mM caffeine. (**C**) MCM2 S108 phosphorylation and FANCD2-Ub levels were measured in cells treated with negative control or ATR-targeting siRNA for 48 h prior to the addition of 4-mM HU for 2 h, and DMSO, 10-μM polyamide **1** or 1-μM polyamide **2** for 36 h.

Since Chk1, Chk2 and RPA2 were not phosphorylated, our results suggested that ATR phosphorylates targets intrinsic to the replication fork to regulate S-phase progression. MCM2 is a component of the replicative helicase and is required for both initiation and elongation phases of DNA replication. MCM2 S108 is phosphorylated by ATR and ATM in response to stalled replication and DSBs (45). This phosphorylation is thought to be an attempt by the cells to promote the firing of local dormant replication origins via Plk1 in order to ensure complete replication (46). We monitored MCM2 phosphorylation for response to polyamide-induced replication stress. Treatment with polyamide **1** or **2** resulted in a time-dependent increase of MCM2 S108 phosphorylation. The level of MCM2 phosphorylation observed after 36-h polyamide treatment was similar to that observed after 2-h HU treatment. Polyamide-induced MCM2 S108 phosphorylation was also inhibited by co-treatment with caffeine (Figure 4B). To determine the contribution of ATR to MCM2 phosphorylation, ATR was knocked down using siRNA prior to polyamide treatment, and similar levels of inhibition were observed as under caffeine treatment (Figure 4C). We also investigated the contributions of ATR and ATM to MCM2 phosphorylation using the small molecule kinase inhibitors NU6027 and KU55933. Both inhibitors reduced MCM2 phosphorylation levels induced by HU or polyamide, with a stronger effect from NU6027 (Supplementary Figure S4A). Together, these observations suggest that ATR is the predominant mediator of polyamide-induced MCM2 phosphorylation.

Recently, the FA/BRCA pathway protein FANCD2 was implicated in a general replisome surveillance mechanism (47). In response to replication stress, FANCD2 undergoes ATR-dependent monoubiquitination (48), which is critical for prolonged localization to chromatin at stalled replication forks (47,49). FANCD2 functions to protect stalled forks from degradation (49) and physically interacts with phosphorylated MCM2 (47), though interaction with MCM2 is not dependent upon monoubiquitination. This led us to search for polyamide-induced monoubiquitination of FANCD2 as a marker of FA/BRCA pathway activation. Treatment with polyamide **1** or **2** caused a time-dependent increase in monoubiquitinated FANCD2 (FANCD2-Ub) (Figure 4A). FANCD2-Ub was present in vehicle-treated samples, which is perhaps a consequence of DU145 cells’ endogenous genomic instability. Surprisingly, inhibition of ATR through the use of caffeine or siRNA both failed to decrease the level of polyamide-induced FANCD2-Ub (Figure 4B and C). In addition, the ATR inhibitor NU6027 increased the fraction of FANCD2-Ub in response to polyamide treatment (Supplementary Figure S4B). These results may be unique to DU145 cells, as ATR knockdown by siRNA has been shown to abrogate FANCD2 ubiquitination in the presence of high levels of replication fork damage caused by 12-h treatment of HU or mitomycin C (MMC) in U2OS cells (50). The ATM inhibitor KU55933 similarly increased FANCD2-Ub levels when co-treated with polyamides, though this result is consistent with previous studies (Supplementary Figure S4B) (51).

Next, we confirmed the functional role of FANCD2 in resisting the toxic effects of polyamides in a model outside of prostate cancer. The FANCD2-deficient fibroblast cell line PD20 complemented with an empty vector exhibited greater sensitivity to polyamide treatment than PD20 cells complemented with a FANCD2-expressing vector (Supplementary Figure S5). Together, these data support the conclusion that MCM2 and FANCD2 participate in the response to polyamide-induced replication stress in addition to ATR.

### Polyamide 1 inhibits T7 gp4A helicase activities in vitro

**Figure 5.**
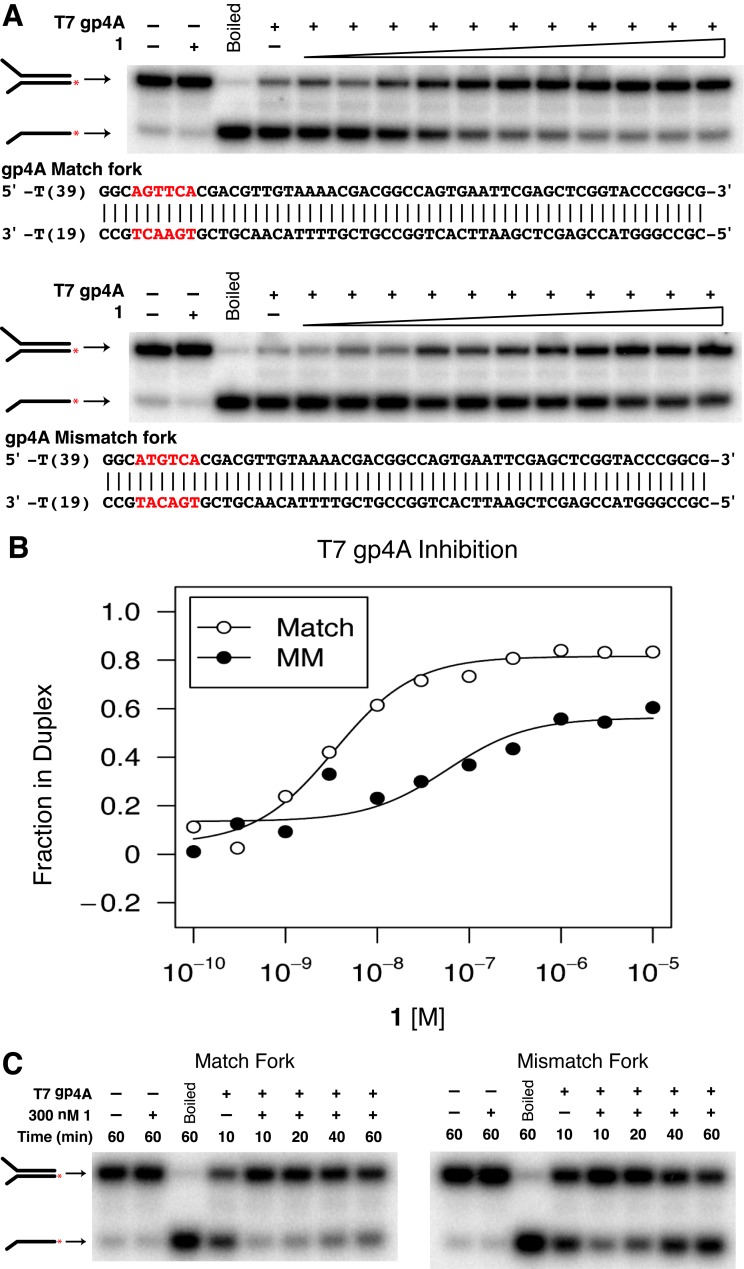
Polyamide **1** inhibits T7 gp4A helicase activity. (**A**) Inhibition of T7 gp4A by polyamide **1** was tested using a forked DNA duplex containing a single match-binding site (top) or no match-binding site (bottom). ^32^P is represented in the cartoon by the red asterisk. Polyamide **1** was added in increasing concentrations (lanes 4–15): 100 pM, 300 pM, 1 nM, 3 nM, 10 nM, 30 nM, 100 nM, 300 nM, 1 μM, 3 μM and 10 μM. (**B**) Graphical representation of gp4A inhibition curves. (**C**) Inhibition of gp4A was then assessed in the time domain by incubating the helicase reactions for increasing amounts of time with either the (left) matched or (right) mismatched substrate.

The results from cell culture experiments suggested a model in which polyamides stall replication forks without causing extensive ssDNA or DNA breaks and that the ATR and the FA/BRCA pathways are activated. The high-affinity DNA-binding properties of polyamides coupled with limited ssDNA formation suggested that polyamides might inhibit unwinding of the replication fork. To test this hypothesis, we determined the ability of polyamide **1** to inhibit DNA helicases *in vitro*. We first studied a strong replicative, hexameric helicase similar to the MCM2-7 complex. Testing both polyamides was deemed unnecessary for this particular study, as both polyamides **1** and **2** have comparable binding affinities *in vitro* as shown by a duplex DNA thermal stabilization assay (Supplementary Figure S6). We used T7 gp4A, the well-studied T7 phage homohexameric replicative helicase (23). We followed unwinding of a forked duplex DNA substrate containing either a single match site or no match sites for polyamide **1** by gel electrophoresis (23). Helicase inhibition was measured by the percent of unwound substrate relative to the mock-treated sample. Incubating polyamide **1** with the substrate containing the match site resulted in effective inhibition of gp4A helicase activity (IC_50_ ∼ 5 nM) (Figure 5A, top). The polyamide was still able to inhibit gp4A helicase activity on the mismatch substrate but required significantly higher concentrations of polyamide (IC_50_ ∼ 335 nM), owing to the sequence specificity of polyamides (Figure 5A, bottom). Similar results were also obtained when using a different class of helicase, *S. cerevisiae* Dna2 (Supplementary Figure S7). These results suggest that the polyamide is not directly interacting with the helicases but acts through DNA binding.

The sequence-specific non-covalent binding nature of polyamides led us to hypothesize that helicase inhibition would not only be stronger at a given concentration when comparing the match to the mismatch substrate but that the enzyme would also show slower unwinding kinetics given the polyamide's longer dwell time at a match site. When using the match substrate, gp4A was unable to unwind as much substrate in the presence of polyamide as the mock-treated sample even when allowed to incubate for longer times (Figure 5C, left). However, gp4A was capable of unwinding the same amount of mismatch substrate in the presence of polyamide as the mock-treated sample when allowed to incubate longer (Figure 5C, right). These data support helicase inhibition as one explanation for how polyamides cause replication stress.

## DISCUSSION

**Figure 6.**
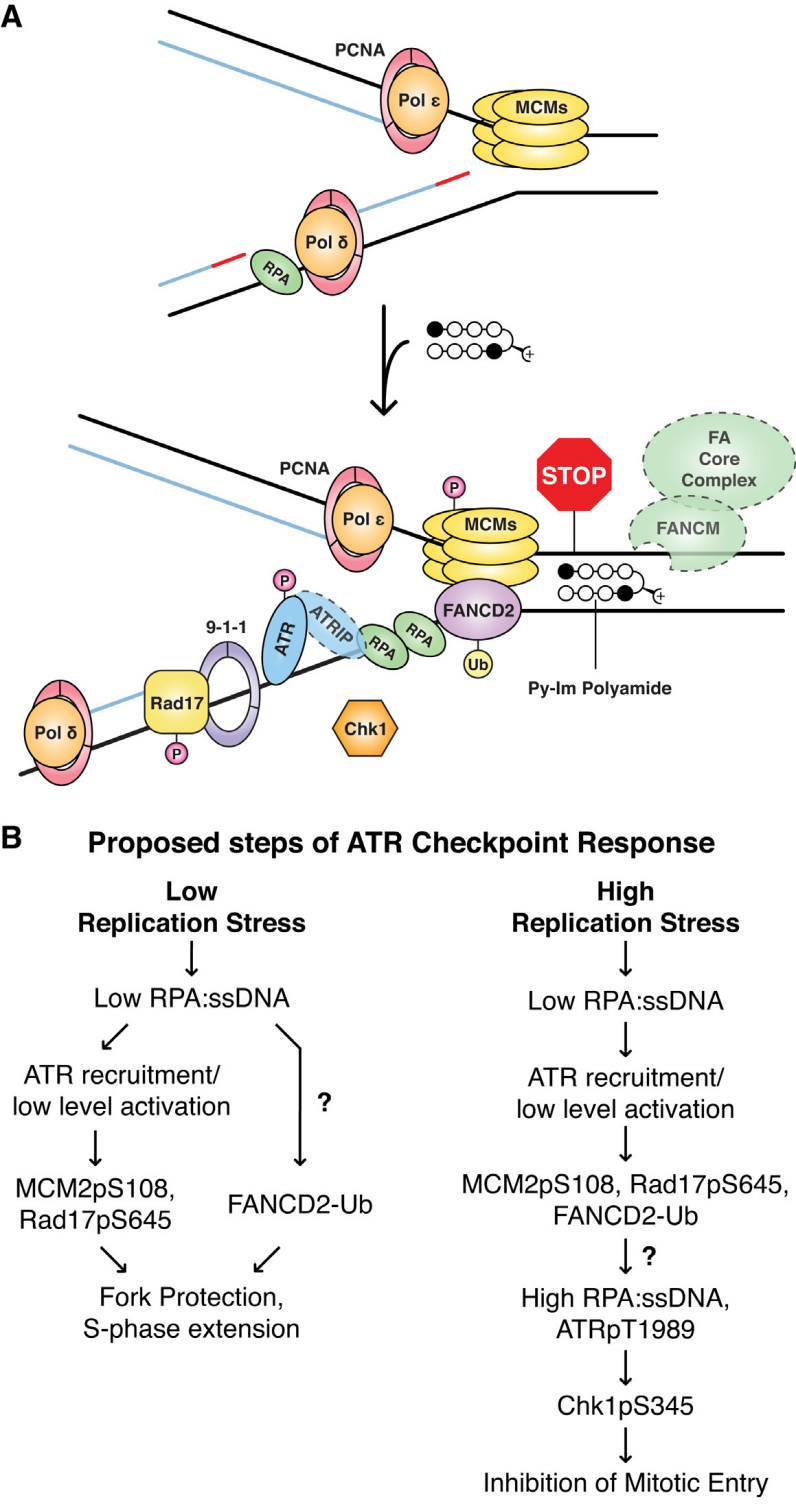
Putative model of Py–Im polyamide-induced replication stress and subsequent ATR-dependent checkpoint response. (**A**) Polyamides bind transiently at match sites throughout the genome, distorting the structure of the helix locally and precluding the progression of the replisome when encountering a fork. Stalled replication fork components that were not investigated directly (except polymerases) are outlined in dashed lines. (**B**) Proposed steps of ATR checkpoint response under low replication stress, such as polyamide treatment, or high replication stress, such as high concentration HU treatment. Our data support the model for stepwise activation of ATR. First, ATR is recruited to chromatin and moderately phosphorylated, leading to MCM2 phosphorylation and Rad17 phosphorylation. FANCD2 is also monoubiquitinated and recruited to chromatin for fork protection. Then, if the stress is sufficiently high, such that replication forks are persistently stalled, ssDNA accumulation and higher ATR T1989 phosphorylation occur, followed by downstream phosphorylation of Chk1 by ATR. What triggers the switch leading to ssDNA accumulation and ATR-Chk1 activation is unclear.

In the present study, we determine that hairpin Py–Im polyamides designed to target the AR:DNA interface are cytotoxic and cause replication stress in androgen-insensitive DU145 cells. Polyamide-induced replication stress causes the accumulation of S-phase cells and PCNA foci, decreased replication, and triggers chromatin loading and activation of ATR. The ssDNA-binding protein subunit, RPA2, and the downstream effector kinase, Chk1, were not phosphorylated in response to polyamide treatment, even at high concentrations and after long incubations. ATR did, however, phosphorylate the MCM helicase subunit, MCM2. In addition, the phospho-MCM2 binding partner and FA/BRCA family member, FANCD2, was monoubiquitinated following polyamide treatment. ATR activation also led to phosphorylation of Rad17, the major subunit of the checkpoint clamp loader. In sum, the polyamide-induced checkpoint response, like that induced by nucleotide depletion, requires the general replisome surveillance pathway involving FANCD2, but does not also require the canonical Chk1 pathway that nucleotide depletion activates to mitigate the stress. Consistent with the DNA helix altering and duplex stabilization properties of polyamides, we showed that polyamides inhibit a hexameric replicative helicase *in vitro* and postulate a model in which non-covalently binding polyamides intermittently preclude replisome progression, resulting in a limited ATR checkpoint response (Figure 6A).

When activated at a stalled replication fork, ATR is critical for protection of the forks from collapse. ATR also suppresses the firing of dormant origins globally, presumably to prevent further replication-associated damage (6). Recently, Koundrioukoff *et al.* (52) reported that ATR could be activated in discrete stages. Low-concentration aphidicolin treatment, which resulted in moderately reduced fork speeds, led to recruitment of ATR and ATR activators to chromatin as well as delayed mitotic entry but did not result in ssDNA accumulation or Chk1 phosphorylation. In addition, low concentrations of aphidicolin did not induce ATM or H2AX phosphorylation. Based on the similarity in checkpoint response to polyamides, we propose that polyamides induce low-level replication stress leading to ATR recruitment and cell cycle delay decoupled from Chk1 activity.

Although the previous work established that activation of the ATR checkpoint response might occur in the absence of downstream Chk1 activation, it did not identify the mediators of fork protection. Our findings implicate ATR-dependent phosphorylation of MCM2 and FA/BRCA pathway activation, as evidenced by monoubiquitination of FANCD2. ATR-mediated MCM2 phosphorylation has previously been shown to recruit Plk1 to stalled forks, which may allow origin firing near the stall for completion of replication (46). FANCD2 has been shown to bind nascent DNA at sites of replication stalling due to nucleotide depletion and, importantly, restrains replisome progression to minimize ssDNA accumulation (47,53). FANCD2 bound to nascent DNA interacts transiently but directly with the MCMs, including phosphorylated MCM2, though this interaction does not depend on monoubiquitination of FANCD2 (47). However, this interaction was shown to depend on ATR activity. It is interesting that polyamide-induced FANCD2 monoubiquitination in DU145 cells was not inhibited upon knockdown of ATR. The current model of FA/BRCA pathway activation, based on studies in U2OS osteosarcoma cells, DT40 chicken B cells and *in vitro* assays, links ATR to downstream FANCD2 monoubiquitination through the phosphorylation of FANCI, a FANCD2 paralog, in the presence of catastrophic interstrand crosslinking damage or long-term treatment with HU (48,50,54). It is possible that replication stress may trigger FANCI phosphorylation by a kinase other than ATR in DU145 cells or that FANCI is an ATR substrate under more severe forms of replication stress. However the FA/BRCA pathway is activated, our results suggest that the FA/BRCA pathway acts in concert with ATR-MCM2 signaling to stabilize replication forks in response to polyamide treatment. The lack of ATR dependence on polyamide-induced S-phase accumulation is also notable, but consistent with published studies in U2OS cells treated with HU (50). Investigating the effects of knockdown of FA family genes on ssDNA formation and cell cycle phase distribution in polyamide-treated cells would be of interest for future studies.

In order to understand how ATR-MCM2 and FA/BRCA activation is related to ATR-Chk1 activation, we compared the checkpoint response induced by low replication stress, such as polyamide treatment, and high replication stress, such as high-concentration HU treatment (Supplementary Figure S8). Both treatments result in MCM2 phosphorylation and FANCD2 monoubiquitination, as well as recruitment of equivalent amounts of ATR and its mediators to chromatin (Figure 2E). However, polyamide treatment resulted in significantly lower levels of ssDNA formation (Figure 2C and D). Our data suggest that polyamide treatment either induces sufficient ssDNA for ATR recruitment or perhaps triggers an alternative or cooperative mechanism to recruit ATR-ATRIP to DNA. The amount of ssDNA is also sufficient for partial ATR activation, as indicated by Rad17 phosphorylation. We hypothesize that only in the presence of higher levels of ssDNA is ATR fully activated and Chk1 phosphorylated, in keeping with the fact that Chk1 phosphorylation depends on the formation of long ssDNA gaps (55). Polyamide treatment also induced much lower levels of ATR T1989 phosphorylation than did HU treatment. This correlates as well with the lack of Chk1 phosphorylation, which requires robust ATR T1989 autophosphorylation (31,32), and is consistent with a model for quantitative regulation of ATR (32). ATR T1989 phosphorylation has actually been shown to be dispensable for ATR recruitment, Rad17 S645 phosphorylation and recovery from transient replication stress (31,32). Based on these data, we conclude that ATR-MCM2 and FANCD2 signaling are sufficient to induce some fork protection. However, ATR-Chk1 cell cycle checkpoint activation requires ssDNA accumulation and extensive ATR T1989 phosphorylation, which is observed under higher replication stress (Figure 6B). While it is unclear what causes ssDNA accumulation and ATR-Chk1 activation, some possible causes are uncoupling of polymerase and helicase, accumulation of excess primers or nascent DNA degradation.

A few studies have shown previously that the FA/BRCA pathway and the ATR-Chk1 pathway serve non-redundant functions and that their signaling mechanisms are separable. In human primary fibroblasts, Chk1 and FANCD2 both contribute to senescence induction but Chk1 is also responsible for persistent cell cycle arrest in response to psoralen treatment (56). Similarly, knockdown of FANCD2 but not Chk1 sensitizes HeLa cells to cisplatin treatment, despite activation of Chk1 (57). Supporting the evidence for their different functions, it has been shown that the canonical ATR activators, Rad17 and TopBP1, are necessary for Chk1 phosphorylation but dispensable for FANCD2 monoubiquitination and FANCI phosphorylation in DT40 cells treated with MMC (58). Conversely, the FA core complex is necessary for FANCD2 monoubiquitination, but is dispensable for Chk1 phosphorylation (58). Also, the interaction of FANCD2 with the MCMs is not dependent on Chk1 activity (47). Thus, the activation of the FA/BRCA pathway but not Chk1 in response to polyamide treatment appears to reflect a level of stress that does not require intervention by Chk1.

Hairpin Py–Im polyamide-induced replication stress causes what appears to be an intermediate state of ATR-dependent checkpoint response. We suggest that this is due to transient inhibition of replisome progression caused by the polyamide's unique high-affinity non-covalent DNA-binding properties. This proposed mode of action distinguishes hairpin Py–Im polyamides from other replication inhibitors such as HU and aphidicolin and will prove useful for further dissociating the S-phase, essential ATR functions from G2 checkpoint functions. Further delineation of the S-phase-specific ATR mediators and effectors involved in protecting replication forks can be determined as distinct from or coordinated with those involved in cell cycle slowing.

## SUPPLEMENTARY DATA

Supplementary data are available at NAR Online.

SUPPLEMENTARY DATA
