# Supplementary material for: Replication stress by Py–Im polyamides induces a non-canonical ATR-dependent checkpoint response
Source: Nucleic Acids Res. 2014 Sep 23;42(18):11546–59. doi: 10.1093/nar/gku866 (PMC4191428; doi:10.1093/nar/gku866)
Supplement: SUPPLEMENTARY DATA [file supp_42_18_11546__index.html]

Replication stress by Py–Im polyamides induces a non-canonical ATR-dependent checkpoint response — Replication stress by Py–Im polyamides induces a non-canonical ATR-dependent checkpoint response — SUPPLEMENTARY DATA 

# Replication stress by Py–Im polyamides induces a non-canonical ATR-dependent checkpoint response

## SUPPLEMENTARY DATA

**Files in this Data Supplement:**

- SUPPLEMENTARY DATA
