# Supplementary material for: Replication stress by Py–Im polyamides induces a non-canonical ATR-dependent checkpoint response
Source: Nucleic Acids Res. 2014 Sep 23;42(18):11546–59. doi: 10.1093/nar/gku866 (PMC4191428; doi:10.1093/nar/gku866)
Supplement: SUPPLEMENTARY DATA [file supp_gku866_nar-01068-h-2014-File008.pdf]

SUPPLEMENTARY DATA FOR

Replication Stress by Py-Im Polyamides Induces a Non-canonical ATR-dependent Checkpoint Response

Thomas F. Martínez[1,†], John W. Phillips[1, †], Kenneth K. Karanja[2], Piotr Polaczek[2], Chieh-Mei Wang[1], Benjamin C. Li[1], Judith L. Campbell[2,*], Peter B. Dervan[1,*]

[1] Division of Chemistry and Chemical Engineering, California Institute of Technology, Pasadena, CA 91125, USA
[2] Braun Laboratories, California Institute of Technology, Pasadena, CA 91125, USA

* To whom correspondence should be addressed. Tel: +1 626 395 6002; Fax: +1 626 683 8753; Email: dervan@caltech,edu. Correspondence may also be addressed to Judith L. Campbell. Tel: +1 626 395 6053; Fax: +1 626 449 0756; Email: jcampbel@caltech,edu

† The first two authors should be regarded as joint First Authors.

		Cytotoxicity IC_{50} values (μM)			
**Cell line**	**AR**	**1**		**2**	
		**72h**	**96h**	**72h**	**96h**
**LNAR**	+++	40±10	36±14	3±1	1.5±0.2
**LNCaP**	+	18±4	7±3	1.8±0.9	0.6±0.2
**DU145**	-	14±4	8±4	1.5±0.5	0.76±0.06

Supplementary Table 1. Summary of cytotoxicity IC_{50} values of polyamides **1** and **2** in AR-overexpressing (+++, LNAR), AR-expressing (+, LNCaP), and AR-negative (-, DU145) cancer cell lines. Cells were treated continuously with polyamides for 72 or 96 h before fixation and staining. Values represent the mean ± S.D. of three replicates.

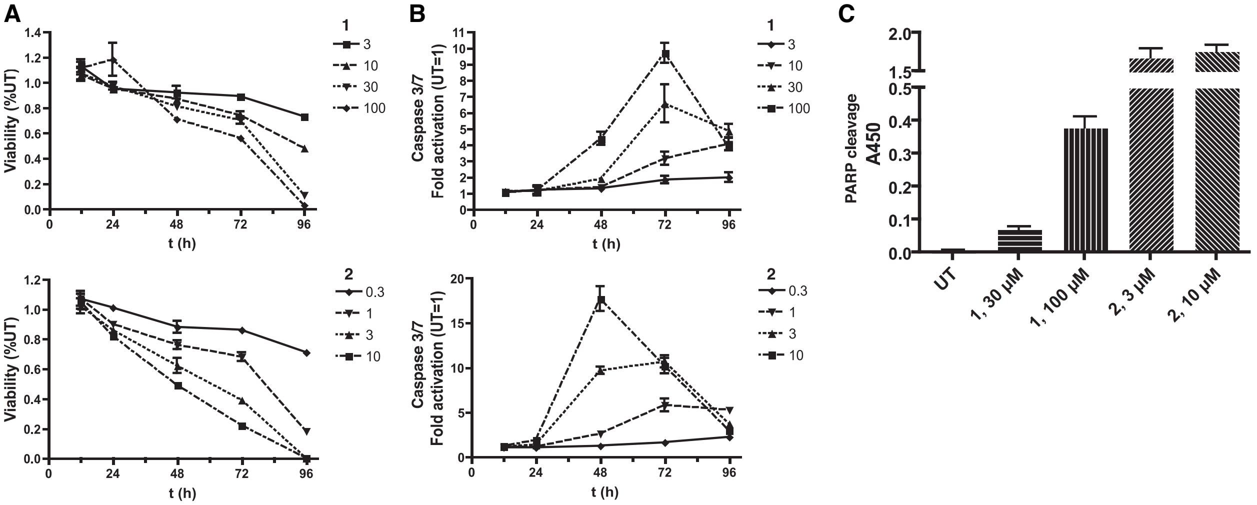

Supplementary Figure 1. Polyamides induce apoptosis in DU145 cells. (**A**) Cell viability assay. Cells were treated in quadruplicate with polyamide **1** (top) or polyamide **2** (bottom) for range of concentrations (µM) for up to 96 h and then assayed for bioreductive capacity with WST-1 reagent. The data are normalized to the untreated condition. (**B**) Caspase 3/7 activity assay. Cells were treated in triplicate with polyamide **1** (top) or polyamide **2** (bottom) for the indicated time and then homogenized in guanidinium lysis buffer containing a pro-luminescent Caspase 3/7 substrate. The data are normalized to the untreated condition. (**C**) ELISA for cleaved PARP formation. Cells were treated with polyamides for 72 h. before assaying the lysates by sandwich ELISA using an HRP-conjugated secondary antibody and a chromogenic substrate. The data are presented as the background-corrected absorbance values at 450 nm. Error bars represent the mean ± S.D. of experiments conducted in triplicate or quadruplicate.

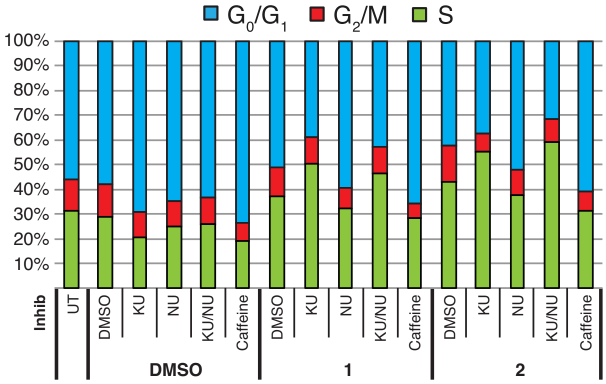

Supplementary Figure 2. Effects of small molecule PI3-kinase inhibitors on polyamide-induced S-phase accumulation. Cell cycle distribution of DU145 cells after 36 h treatment with DMSO, 10 μM polyamide **1**, or 1 μM polyamide **2** in the presence of 2 mM caffeine, 10 μM KU55933 (KU, ATM inhibitor), or 10 μM NU6027 (NU, ATR inhibitor) as measured by single-color flow cytometric evaluation of propidium iodide stained cells. When both KU and NU were added together with DMSO or polyamide, only 4 μM of each inhibitor was used to reduce toxicity.

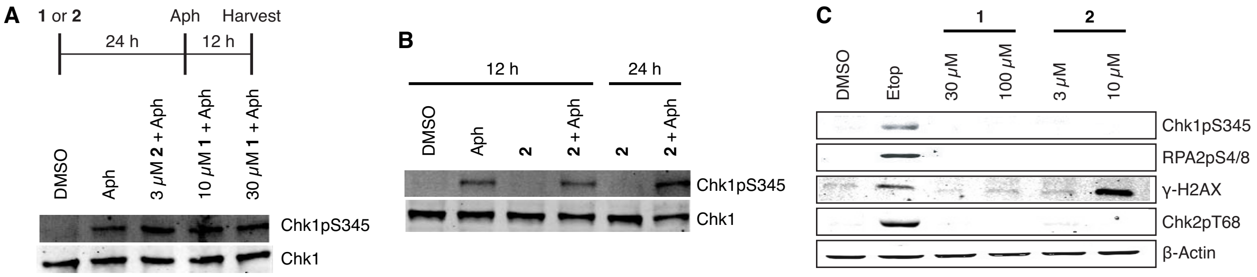

Supplementary Figure 3. High concentration polyamide treatment does not does not inhibit aphidicolin-induced Chk1 phosphorylation. (**A**) Immunoblot of Chk1pS345 after the treatment with polyamides followed by aphidicolin. DU145 cells were treated with DMSO, 10 μM polyamide **1**, or 1 μM polyamide **2** followed by the addition of 10 μg/mL aphidicolin (Aph) after 24 h. Cells were harvested after 36 h total incubation. (**B**) Immunoblot of Chk1pS345 after simultaneous treatment of DMSO or 3 μM polyamide **2** plus 10 μg/mL aph for 12 or 24 h. (**C**) Immunoblot of S-phase checkpoint and DNA damage response proteins, Chk1pS345, RPA2pS4/8, Chk2pT68, and γ-H2AX in DU145 cells after 18 h treatment with DMSO, 30 μM etoposide, polyamide **1**, or polyamide **2** at the indicated concentrations.

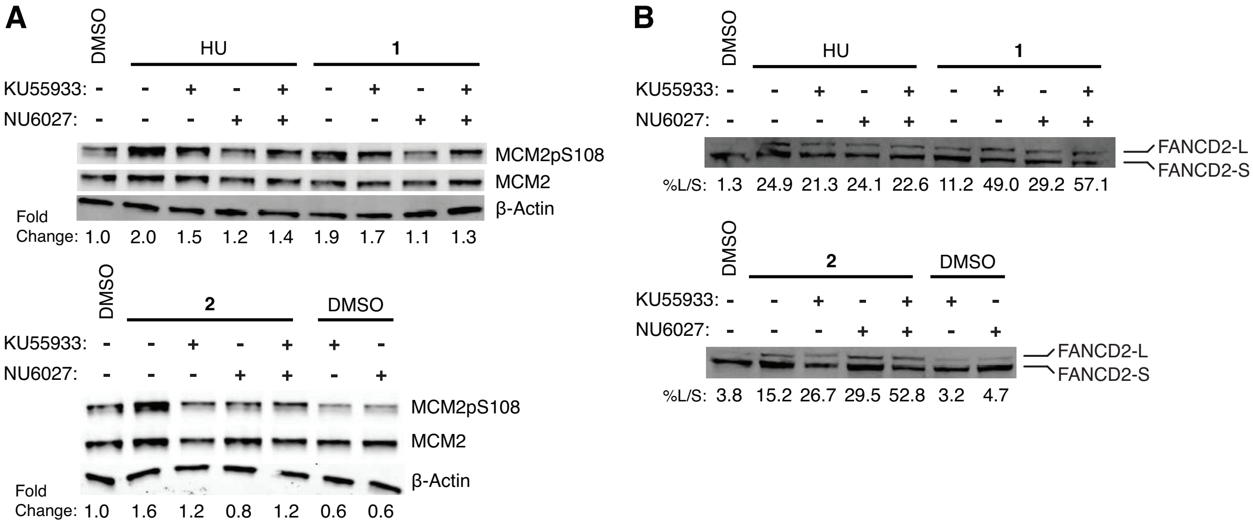

Supplementary Figure 4. Effects of ATM- and ATR-specific small molecule inhibtors on polyamide-induced MCM2 S108 phosphorylation and FANCD2 monoubiquitination. (**A**) MCM2 S108 phosphorylation levels were measured in DU145 cells treated with 4 mM HU for 2 h, and DMSO, 10 μM polyamide **1** or 1 μM polyamide **2** for 36 h in addition to 10 μM KU55933 (KU, ATM inhibitor), 10 μM NU6027 (NU, ATR inhibitor), or both KU and NU. Only 4 μM KU and 4 μM NU were used when both inhibitors were added together to reduce toxicity. (**B**) FANCD2-Ub levels were measured in selective kinase inhibitor-containing lysates. Monoubiquitination was estimated by normalizing the band intensity of the large molecular weight monoubiquitinated FANCD2 band (FANCD2-L) to the low molecular weight non-ubiquitinated FANCD2 band (FANCD2-S).

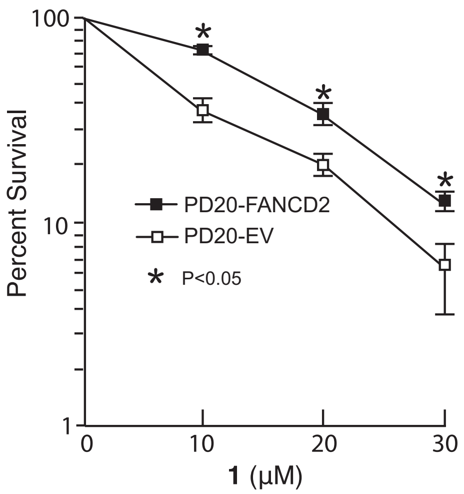

Supplementary Figure 5. FANCD2 increases cell survival after exposure to polyamide **1**. PD20 cells complemented with empty vector (PD20-EV) or FANCD2 (PD20-FANCD2) were treated with the indicated concentrations of polyamide **1** for 36h and assayed for survival after 14 days. Error bars indicate mean ± SEM for n=3 independent experiments. Unpaired T-tests were performed at *P<0.05.

A

```
5'-TCGC AGAACA GCGA-3'
3'-AGCG TCTTGT CGCT-5'
```

T_m = 61.7 (±0.4) °C

	T_m (°C)	ΔT_m (°C)
**1**	76.7 (±0.1)	14.9
**2**	76.2 (±0.2)	14.4

B

```
5'-GT AGAACA GCGACC-3'
3'-CA TCTTGT CGCTGG-5'
```

T_m = 61.4 (±0.3) °C

	T_m (°C)	ΔT_m (°C)
**1**	74.8 (±0.1)	13.3
**2**	74.4 (±0.2)	12.9

C

```
5'-C AGAACA GCAGTCG-3'
3'-G TCTTGT CGTCAGC-5'
```

T_m = 61.3 (±0.5) °C

	T_m (°C)	ΔT_m (°C)
**1**	73.5 (±0.4)	12.1
**2**	74.2 (±0.3)	12.8

Supplementary Figure 6. Py-Im Polyamides stabilize duplex DNA regardless of match site position in the duplex. DMSO or 4 μM polyamide was incubated with 2 μM 14 bp duplex DNA containing only a single 5'-WGWWCW-3' binding site positioned either 4 bps (**A**), 2 bps (**B**), or 1 bp (**C**) from the edge and a melting curve was measured using DNA hyperchromicity (59). The average melting temperature and standard deviation were calculated from four replicates.

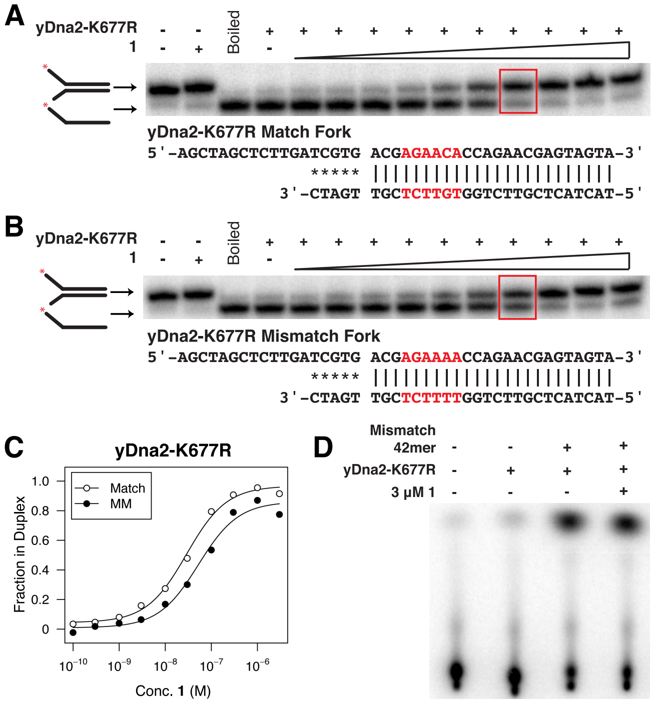

Supplementary Figure 7. Polyamide **1** inhibits helicase activity of *S. cerevisiae* Dna2 nuclease dead but helicase active mutant (yDna2-K677R) but not its ATPase activity. Inhibition of yDna2-K677R by polyamide **1** was tested using a forked DNA duplex containing either one match-binding site (**A**) or no match-binding site (**B**). ^{32}P is represented in the cartoon of the substrate by the red asterisk. Polyamide **1** was added in increasing concentrations (lanes 4-14): 100 pM, 300 pM, 1 nM, 3 nM, 10 nM, 30 nM, 100 nM, 300 nM, 1 μM, 3 μM. (**C**) Graphical representation of yDna2-K677R inhibition curves. (**D**) 3 μM polyamide **1** was incubated with the single-stranded mismatch DNA oligomer and yDna2-K677R to assess whether polyamide **1** can inhibit yDna2-K677R ATPase activity.

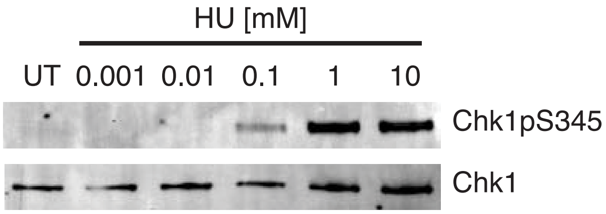

Supplementary Figure 8. Dose-dependent increase in hydroxyurea (HU)-induced Chk1 S345 phosphorylation. Chk1 S345 phosphorylation was measured in DU145 cells treated with increasing doses of HU for 2 h. Chk1 S345 is maximally phosphorylated at 1 mM or higher HU.

REFERENCES

59. Dose, C., Farkas, M.E., Chenoweth, D.M. and Dervan, P.B. (2008) Next generation hairpin polyamides with (R)-3,4-diaminobutyric acid turn unit. *J. Am. Chem. Soc.*, 130, 6859-6866.
